# Empathy in patient care: from ‘Clinical Empathy’ to ‘Empathic Concern’

**DOI:** 10.1007/s11019-021-10033-4

**Published:** 2021-07-01

**Authors:** Clarissa Guidi, Chiara Traversa

**Affiliations:** University of Groningen, Groningen, The Netherlands

**Keywords:** Patient care, Clinical empathy, Taxonomy, Empathic concern, Engaged curiosity, Narrative medicine

## Abstract

As empathy gains importance within academia, we propose this review as an attempt to bring clarity upon the diverse and widely debated definitions and conceptions of empathy within the medical field. In this paper, we first evaluate the limits of the Western mainstream medical culture and discuss the origins of phenomena such as *dehumanization* and *detached concern* as well as their impacts on patient care. We then pass on to a structured overview of the debate surrounding the notion of clinical empathy and its taxonomy in the medical setting. In particular, we present the dichotomous conception of clinical empathy that is articulated in the debate around *cognitive empathy* and *affective empathy*. We thus consider the negative impacts that this categorization brings about. Finally, we advocate for a more encompassing, holistic conception of clinical empathy; one that gives value to a genuine interest in welcoming, acknowledging and responding to the emotions of those suffering. Following this line of reasoning, we advance the notion of ‘empathic concern’, a re-conceptualization of clinical empathy that finds its source in Halpern in Med Health Care Philos (2014) 17:301–311 *engaged curiosity*. We ultimately advance Narrative Medicine as an approach to introduce, teach and promote such an attitude among medical trainees and practitioners.

## Introduction

In the last thirty years, the notion of empathy has gained great attention within academia. Commonly defined as “feeling as others” (Hein and Singer 2008, p. 157), the value of empathy has been up for debate. If some emphasize the importance of empathy as the essential tool for achieving a better civil society (Nussbaum 1996) and dealing with prominent global issues such as climate crisis (Rifkin 2009), others believe that empathy can “motivate cruelty and aggression and lead to burnout and exhaustion” (Bloom 2017, p. 24).

Depending on the field of research, the notion of empathy has acquired different connotations and meanings (Cuff et al. 2016). In the medical field the definition of empathy has been widely debated among scholars; whereas some hold that clinical empathy is limited to a mere cognitive understanding of others’ emotional states (Finset 2010), others believe that clinical empathy should reflect the human purpose of medicine (Hardy 2017; Halpern 2014).

The relation between empathy and healthcare is particularly prominent in ‘Narrative Medicine’, an approach which focuses on promoting the importance of storytelling and on facilitating the empathic encounter between health practitioner and patient.

Twelve years since the establishment of the Master of Science in Narrative Medicine at Columbia University, we decided to explore the current state of empathy in the medical field and particularly in the caregiver-patient interaction.

Our aim is to provide a systematic overview of the debate on the topic, and to offer our views on the notion of ‘clinical empathy’ understood as “the ability to *observe* emotions in others, the ability to *feel* those emotions, and finally the ability to *respond* to those emotions” (Finset 2010, p. 4, our italics). Ultimately, we support the conception of clinical empathy as a result of a “genuine, emotionally engaged *interest* in learning more about the complexity of the patient’s point of view” (Halpern 2014, p. 308, our italics); we thus advance the notion of ‘empathic concern’ as a conceptual substitute for clinical empathy.

### Relevance

In accordance with Haque and Waytz (2012), we believe that the working of the current medical system can be detrimental to the well-being of both health professionals and patients. More specifically, the current medical approach has been repeatedly criticized for becoming a narrow and rigid system that does not honor the subjective experience of human suffering (Charon 2006). Rather, the longstanding adherence and reliance on a specific conception and application of the scientific method has, in the long run, translated itself into a cold, detached and dehumanized healthcare. Objectivity, emotional detachment and detached concern appear to be all-encompassing and thereby leave no or insufficient space for the human dimensions of illness. This phenomenon has shown to cause negative consequences for health practitioners and patients alike (Eknam and Krasner 2017).

As mentioned above, the mainstream medical culture is characterized by its reliance on objectivity, emotional detachment and detached concern (Coulehan and Williams 2001 cited in Shapiro 2011). In this paper, we will further explore how such concepts can be limiting rather than beneficial to the medical practice. Mere scientific rigor within the medical field has led to overlooking the human aspects of disease as well as its multidimensionality (Johna and Rahman 2011); the latter encompassing the psychological, social and moral dimensions of suffering. In this context, the distinction between disease and illness, defined as the “innately human experience of symptoms and suffering” (Johna and Rahman 2011, p. 92), is ignored and disregarded (Reiss 2010).

Following this line of reasoning, we speak of detached concern as the most aspired ideal for medical professionalism, as it expresses the capacity of being concerned about something without being emotionally involved (Aring 1958, Blumgart 1964 cited in Halpern 2014).

Detached concern has become an attitude that reflects a commitment to eradicate disease rather than a genuine interest in providing care. This has led to a widespread tendency to overlook ‘clinical empathy’, here defined as “the ability to *observe* emotions in others, the ability to *feel* those emotions, and finally the ability to *respond* to those emotions” (Finset 2010, p. 4, our italics).

As we will see, multiple studies have shown that clinical empathy brings benefits to patients, medical students and health practitioners. In concrete terms, positive clinical outcomes for patients (AAMC 1998 cited in Ekman and Krasner 2017; Gleichgerrcht and Decety 2013 cited in Hooker 2015; Hojat et al. 2011, 2013; Rosenthal et al. 2011) include therapeutic effectiveness, improvement in physiological responses and shorter hospitalization. Benefits that not only improve patients’ well-being (Stepien and Baernstein 2006; Bikker 2005; MacPherson et al. 2003; Coulehan et al. 2001; Vermeire et al. 2001; Suchman et al. 1993 cited in Chen et al. 2012) but also bring about economic advantages (Ekman and Krasner 2017; Rosenthal et al. 2011; Shanafelt et al. 2009 cited in Reiss 2010; Hojat 2009).

Furthermore, as we will touch upon, correlated benefits for health practitioners include an increase in professionals’ personal well-being and job satisfaction (Shanafelt et al. 2012; AAMC 1998 cited in Ekman and Krasner 2017; Larson and Yao 2005 cited in Hooker 2015; Rosenthal et al. 2011; Hojat 2009) together with a decrease in malpractice claims and patients’ litigations (Rosenthal et al. 2011; Hojat 2009). Additionally, higher levels of empathy have been correlated with a decrease in stress symptoms for both medical trainees and physicians (Shanafelt et al. 2009 cited in Reiss 2010).

### Structure

The article begins by reviewing the limits of the current medical culture. Here, we first consider the origins of the phenomena of dehumanization and detached concern by looking at the American medical tradition and culture, greatly shaped by the teachings of Abraham Flexner. Secondly, we analyze the educational consequences of Flexner’s model. More specifically, we look at the decline of empathy in medical education and training, which rather reinforces a “tacit commitment to an ethic of detachment, self-interest and objectivity” (Coulehan and Williams 2001 cited in Shapiro 2011; p. 275).

We then pass on to explore the different conceptions of empathy within the medical setting and shed light upon the debate regarding the topic and its impact on the medical culture. While some scholars support what has been defined as cognitive empathy, others hold that affective empathy is also essential for the quality of patient care and the healthcare system in general (Hardy 2017; Gair 2012; Gallagher 2003 cited in Halpern 2014; Coplan and Goldie 2011; Stueber 2006 cited in Hooker 2015; Halpern 2011; Shapiro 2011; Garden 2009).

Within this context we argue that the *dichotomous* vision that prevails within such debate has led to an excessively rigid taxonomy. We try to overcome this by presenting the views of scholars that support a more inclusive and holistic definition of how empathy in the clinical setting should be understood. Following this discussion, we look at the clinical, social and economic benefits of this latter interpretation of clinical empathy for both patients and healthcare professionals.

This being said, we attempt to overcome potential downfalls of the rigid taxonomy surrounding the term ‘empathy’, whereby empathy is dichotomously divided into ‘cognitive’ and ‘emotional’ empathy. This is why we advance the notion of *‘empathic concern’*, where the focus rather lies on the notion of ‘concern’ that configures itself as a genuine interest towards the other and that results from ‘engaged curiosity; an attitude that gives value to the act of welcoming and acknowledging the complexity in the experience of those suffering (Halpern 2011).

Accordingly, empathic concern bears both the more cognitive perspective taking as well as the emotional engagement and responsiveness dimensions of empathy. This is how a mere commitment to heal can translate into a *validation* of the patient’s suffering and needs. In this sense, clinical empathy should be understood as empathic concern and as a result of ‘engaged curiosity’. We ultimately advance Narrative Medicine as an approach to introduce, teach and promote such an attitude among medical trainees and practitioners.

## Limitations of mainstream medical culture

Current Western medical culture is known for its scientific rigor and its emphasis on objectivity and emotional detachment. This culture has led the Western medical system to become highly dehumanized. The origin of dehumanization in the North-American medical tradition can be traced back to the twentieth century, and partly to Abraham Flexner (1886–1959), who went down in history for his reform of medicine and higher education in the United States.

Flexner believed that the proper goal of medicine was to “attempt to fight the battle against disease” (Flexner 1912, p. 23 cited in Johna and Rahman 2011). In fact, he argued that the practice and the development of medicine merely depended on those rigorously trained in natural sciences. Accordingly, Flexner believed that clinicians had to be “impregnated with the fundamental truth of biology” (Flexner 1912, p. 23 cited in Johna and Rahman 2011), thus disregarding all human aspects of disease: the psychological, social and moral dimensions of suffering. Flexner thus ignored the distinction between disease and *illness*; the latter being defined as the “innately human experience of symptoms and suffering” (Johna and Rahman 2011, p. 92).

As medicine progressively relied upon objectivity and scientific rigor, it also sacrificed the subjective human experience of suffering. Illness became a “set of symptoms” (Finset 2010) that affected “bodies, organs and tissues” (Lewis 2011 cited in Johna and Rahman 2011). Still today, medicine tends to overlook patients’ humanity, and rather regards them as a cluster of organs, which display a series of biological and chemical processes. The latter are legitimately rooted at the base of medical training and of clinical practice. However, in accordance with a long tradition of scholarship, we believe that the afore-mentioned emphasis on a mere scientific approach has brought about a medical culture that pays insufficient attention to the value of understanding and honoring the human body and the person as a whole (Haque & Waytz 2012; Reiss 2010).

It is in this sense that we see the current medical approach as a narrow and rigid system; the symptoms one manifests tend to be medically suppressed rather than being taken as tools to *deepen* and *investigate* the etiology of a particular symptom or illness. The body has been separated from the mind and the self; it is in fact cut out of the picture, thus producing a dichotomy in which the physical body-world is deprived of the mental and spiritual spheres. This separation leads to the dehumanization of the ill, whose disease becomes more important than the person themselves.

The establishment of the above-mentioned medical culture, to some extent initiated by Flexner’s reform, deeply shaped the mainstream clinical practice and patient care. In fact, the latter are characterized by the so-called ‘detached concern’, a notion that originated between the 1950s and 1960s (Aring 1958; Blumgart 1964 cited in Halpern 2014). Since then, detached concern has been considered as the most coveted ideal for medical professionalism and its enactment has translated into a widespread tendency to overlook the value of a “genuine, emotionally engaged interest in learning more about the complexity of the patient’s point of view” (Halpern 2014, p. 308).

Accordingly, sociologists identify the loss of the human sphere in medicine and the de-personalization of care as two social phenomena that originated in the 1960s. As objectification, together with commodification and standardization, became the reasoning behind medical achievements, healthcare began to deny the significance of meaning, interest and compassion. In this context, objectification exemplifies and upholds medicine’s commitment to the mechanistic Cartesian mind–body divide, a tendency to transform the subjective/social into the objective/biological (Scheper et al. 1987).

In recent decades, some academics have started to point out the fact that medical education is indeed “overvaluing scientific measurements and undervaluing subjective experiences” (Reiss 2010, p. 1604); something that greatly impacts the quality of healthcare. In fact, illness is not only physical and reducible to biological dysfunctions; it is rather *multidimensional* (Johna and Rahman 2011). As such, the ill person can be disempowered due to bodily impairment, lack of the necessary knowledge and hope to take rational steps towards recovery, loss of some degree of autonomy and the alteration of self-image and identity due to such circumstances (Pellegrino 1981 cited in Johna and Rahman (2011). Likewise, patients are not just bodies, organs and tissues; as any other human being, they “live meaning-centered lives, and they have complicated emotional and historical relationships with their bodies” (Lewis 2011, p. 92 cited in Johna & Rahman 2011).

Nevertheless, the medical system often does not recognize the complex and unique human capacities and resources of patients. As Michel Foucault suggested, medical institutions and the scientific disciplines of modernity are characterized by an objectification of bodies aimed at turning humans into subjects and objects of knowledge (Foucault 1973–1978 cited in Timmermans 2009). In this light, medicine has become a science for science’s sake, leaving behind its original human-centered purpose. This translates to a generalized shortage of empathy in patient care. In fact, medical practitioners are trained to embrace the notion of detached concern and reject engaged human connection.

The above-mentioned principles that characterize the current Western medical culture are also reinforced by and through medical education. Although in recent years, there have been attempts to reintroduce the values of compassion and empathy in the medical training, the medical establishment still relies on a “tacit commitment to an ethic of detachment, self-interest and objectivity” (Coulehan and Williams 2001 cited in Shapiro 2011, p. 275). The medical culture, together with its fear and rejection of human emotions, thus contributes to shaping a medical training that perpetuates “emotional detachment” and “clinical neutrality” (Hojat et al. 2009 cited in Reiss 2010). Correspondingly, physicians learn to feel uncomfortable admitting error. In this regard, studies show that when error occurs, caregivers tend to feel guilty and inadequate (Gallagher et al. 2003 cited in Halpern 2014).

Medical training is based on a positivist worldview that promotes the objectification of patients; the latter can lead to a feeling of alienation from rather than empathy towards the patient, on the part of the doctor (Crandall and Marion 2009 cited in Shapiro 2011). Accordingly, the cultural norms that shape medical education expect aspiring doctors to conceal emotion (Shapiro 2011). Thereby, the most widely accepted conception of medical professionalism is one in which physicians “respond to the suffering of patients with objectivity and detachment” (Coulehan 2009a, b cited in Shapiro 2011).

Overall, medical pedagogy promotes detached concern, which in turn devalues the importance of subjectivity, the role of emotions and the healing power of relationship and solidarity (Coulehan 2005 cited in Shapiro 2011). According to several studies (Bellini and Shea 2005; Hojat et al. 2004; Bellini et al. 2002 cited in Chen et al. 2012), detached concern becomes prominent during the third year of medical training; when students become interns and first enter the professional world. The third-year decline of empathy, partly due to actual contact with suffering, death and deficiency in training in engaging emotions has also been correlated to students’ inexperience in modulating their emotional states in the context of demanding environments (Hojat et al. 2009 cited in Reiss 2010).

## Empathy in the medical setting

In the last two decades the notion of empathy has gained remarkable attention within the academic world. Generally speaking, outside the medical setting, empathy is defined as the ability to ‘feel with’ another person or to put oneself into someone else’s shoes (Halpern 2014).

In the medical setting, definitions of empathy are still widely debated; scholars commonly agree that empathy is distinguished from sympathy. The latter is usually understood as the action of ‘feeling for’ another person; in other words, caring about the patient.

According to some (Trevithick 2005; Boulton 1987 cited in Gair 2012), empathy is understood as ‘feeling with’ the patient. Others, instead, define empathy as the cognitive skill that allows the practitioner to understand the patient. Those who follow this latter line of reasoning create a divide between the cognitive ability to *understand* the other (belonging to their notion of cognitive empathy) and the affective dimension of *feeling for* (which they relegate to sympathy), *and with* the other (which they relate to emotional empathy). In any case, there is a tendency among scholars to distinguish between two main categories of empathy: cognitive empathy and affective empathy.

### Cognitive empathy

‘Cognitive empathy’ is defined as the ability to perceive emotions in others and as the capacity to attribute mental states to them. In other words, it can be understood as an equivalent of the ‘Theory of Mind’ phenomenon (Schulte-Ruther et al. 2007 cited in Finset 2010). This mental process is reached through the so-called ‘Simulation Theory’. According to this theory we understand the mental states of others by simulating them and it is our very ability to see the world from the other’s point of view that allows us to gain an understanding of the other’s experiences of the world (Gladstein 1983; Rushton 1980 cited in Pedersen 2008). This notion of empathy excludes any *emotional* engagement.

The emphasis on the cognitive dimension of empathy makes this notion compatible with the predominant medical culture; one that values detachment, objectivity, and standardization. Thereby, this notion of empathy has become predominant in the context of medical care. In fact, it is believed that it allows physicians to maintain a professional distance from patients, to make objective clinical decisions and to regard patients as equally deserving of care (Coulehan 1995; Roter et al. 1997 cited in Halpern 2011).

Nevertheless, cognitive empathy has been criticized for numerous reasons. In fact, according to Garden (2009), this type of empathy brings about the risk of obscuring rather than shedding light on patients’ experience of illness. This is due to the mentioned simulation process; in fact, “when the physician simulates the patient, the physician’s understanding is limited to what the physician can imagine about the patient’s experiences” (Hardy 2017). This is referred to as “the diversity problem” pointed out by Gallagher (2012), by which the clinician’s understanding of what a patient is going through is limited to what caregivers themselves have experienced. In this sense, it is believed that a shared experience or “common wound” is necessary for empathic understanding (Gair 2012).

According to Shapiro (2011), the strong emotional suppression that characterizes cognitive empathy, and which tends to arise when dealing with death, disability, medical error, and mortality, can lead to exhaustion and burnout; making this vision of empathy less sustainable than what the medical community has upheld. Moreover, according to Hooker (2015), when physicians employ a parallel understanding, they neglect not only the fundamental differences between themselves and the patient, but also the distinction between each individual patient. Lastly, Hardy (2017) holds that the use of cognitive empathy reinforces and upholds the objectification of the patient, by which the individual is still being treated as an *object* for observation and judgment, rather than a *subject* with whom physicians interact.

### Affective empathy

On the other hand, ‘affective empathy’ is described as the emotional engagement that occurs when confronted with the suffering of another person. As such, this form of empathy is disregarded by the medical community and the mentioned medical culture. In fact, it is common belief that emotional empathy relies on “untrustworthy feelings” (Halpern 2014). Despite this attitude, there is growing support of the idea that affective empathy facilitates the recognition of the emotional state and/or predicament of the patient. Additionally, emotional engagement is seen as beneficial as it can kindle the desire ‘to heal’ in physicians; thus, moving beyond the sense of duty to simply eradicate disease.

However, due to a longstanding fear of over-identifying with patients, emotional engagement itself has been identified as one of the main risk factors for the loss of objectivity and the manifestation of emotional distress (Halpern 2001). As we have seen, objectivity is considered the bedrock in most clinical circumstances and interventions; for instance, in the case of a difficult diagnosis and/or when invasive treatment is necessary (Halpern 2014). In fact, it is believed that emotions undermine the ability to achieve successful medical outcomes in delicate circumstances (Coulehan 1995 cited in Halpern 2011).

As already noted, affective empathy is furthermore considered to lead to emotional burnout. This apprehension has determined the implementation of detached concern, shaping decades of medical education and training, which, still to this day, overlooks the individual’s experience of illness. Given the established association between emotional engagement and the occurrence of distress and/or burnout on behalf of physicians (Fox et al. 2009; Alma & Smaling 2006; Astell 2004; Figley 2002 cited in Gair 2012), it has been suggested that emotional down-regulation may be necessary (Reiss 2010 cited in Halpern 2011).

Finally, the attempt to avoid burnout has led to the formulation of an idealized version of empathy on the part of the doctor, “one in which they suppress personal emotions yet are motivated by an altruistic yet ‘detached’ concern for patients” (Halpern 2014, p. 301).

## Debate on the definition of empathy

The heated discussion concerning the above-mentioned divide has led scholars and the medical community to attempt to identify the best type of empathy to call upon in the clinical setting as well as to speculate whether an integration of cognitive and affective empathy may be neurologically possible.

In relation to the first point of debate, for the reasons mentioned above the mainstream medical culture has privileged cognitive empathy over emotional empathy and has established the former as the most suitable type to employ within the clinical setting. However, studies support the idea that both the cognitive and the affective dimensions of empathy are valuable. In fact, as stated by Coplan and Goldie (2011) as well as Stueber (2006), empathy is all about *understanding* the patient. This understanding, however, is not merely cognitive but also involves a genuine emotional response that fully recognizes the patient.

Hence, according to Shapiro (2011), we should not dismiss the emotional component of empathy based on the misconception that emotional engagement is dangerous in-patient care. In fact, Shapiro suggests that, contrary to the dominant belief, purely cognitive empathy without a balanced amount of emotion brings about the risk of making the medical encounter over-operationalized and excessively codified. In other words, “a richer clinical empathy, involving emotional *and* cognitive empathy, makes for more effective medical care.” (Halpern 2011, p. 229, our italics).

Furthermore, in relation to Halpern’s statement (2011), it has been reported that patients are considerably hungry for and deeply desirous of being understood and recognized (Broyard 1993 cited in Hooker 2015). Keeping this in mind, the importance of combining the two facets of empathy is valuable not only to achieve effective medical outcomes, but also to validate and dignify the human needs of patients.

Coming to the second point: given the importance of the co-existence of the discussed cognitive and affective aspects of empathy, scientists have investigated the neurological possibility of the two co-occurring. Research in psychology has tended to assume incompatibility, according to which the two dimensions of empathy are mutually exclusive. The result has been setting one against the other (Zaki et al. 2008 cited in Halpern 2014). Similarly, neuroscientific studies have shown that there are distinct brain pathways in the empathic response. In fact, it appears that different parts of the brain are activated depending on cognitive versus affective cues (Decety 2011 cited in Halpern 2014).

Nonetheless, according to Halpern, these conclusions do not imply that the mentioned brain pathways do not coordinate in real life settings. On this line of reasoning, Zaki et al. (2008) suggests that it is likely that in naturalistic settings they interact in complex ways. In fact, real life interactions involve multimodal cues**,** in contrast to the pathways of separate responses that usually make use of written word narratives versus facial expressions**.**

Thereby, which pathway (and thus type of empathy) one uses depends and varies according to the context. In the medical setting, this translates into an empathic response that is elicited by both verbal and non-verbal cues is elicited by both verbal and non-verbal cues (Halpern 2014).

## Impact of the debate on medical culture

Influenced by the medical culture and its view of cognitive and affective empathy as being mutually exclusive, the medical research community has tried to scientifically prove the need to privilege cognitive empathy over its emotional counterpart. To this regard, in a study conducted by Decety (2010) it was reported that physicians’ brains showed more brain activity than non-physicians in the regions involving executive control and self-regulation; at the same time, they displayed a down-regulation of the brain regions involved in emotional responsiveness. Additionally, another study (Neumann et al. 2011 cited in Halpern 2014) showed that medical students who reported more emotional discomfort in response to their patients’ distress had lower scores in cognitive empathy during their training.

These findings have led some to wonder if medical students with more emotional empathy will lack its more accepted counterpart, that is cognitive empathy. Following this line of reasoning, others have suggested that it is vital for physicians to use cognitive empathy when communicating with patients about painful procedures and that it is fundamental to use cognitive empathy to ensure that the patient’s needs are being adequately addressed (Halpern 2014).

However, the above-mentioned studies overlook important distinctions between self-related anxiety and the emotional distress that may derive from the affective resonance with others; something that still needs further investigation. In fact, as Halpern (2014) suggests, while some physicians may respond with emotional distress when interacting with patients, others may react with a genuine prompt to heal. This calls for further investigation.

## Clinical empathy

As we have seen, there is a dichotomous vision that prevails in the medical culture to the point that medical research has tried to prove the mutual exclusivity between cognitive and affective empathy. Overall, the competing aspect of the debate around the two conceptions of empathy results in creating an excessively rigid taxonomy. In order to overcome such difficulties, scholars have advanced a more inclusive and holistic definition of what empathy in the clinical setting should really entail.

Despite being identified with cognitive empathy, the notion of clinical empathy goes beyond the mere cognitive understanding of the other’s emotions. ‘Clinical empathy’ has been defined as “the ability to *observe* emotions in others, the ability to *feel* those emotions, and finally the ability to *respond* to those emotions” (Finset 2010, p. 4, our italics).

As a multidimensional ability, clinical empathy branches out in three distinct components: a cognitive component, an emotional component and an action component. If the first one is the mental process through which “the physician ‘enters’ the perspective of the patients”, the emotional component allows the practitioner to “[put] himself or herself in the place of the patient”. Finally, the action component, whereby the physician “communicates understanding by checking back with the patient” (Coulehan et al. 2001; Hojat et al. 2001 cited in Chen et al. 2012; Johna and Rahman 2011 cited in Finset 2010).

To this regard, it has been noted by Alma and Smaling (2006) that the empathic communicative response is not merely expressed through words. It can also be non-verbal, including tone of voice, facial expressions, body posture, and natural gestures (Gair 2012). This view is shared by Haslam (2007), who states that “empathy is not only a compassionate appreciation of the patient’s emotions, but also [includes] an expression of that awareness to the patient” (cited in Gair 2012, p. 135). This comprehensive vision of empathy translates to the affective capacity to “feel with” the suffering of the patient and the cognitive ability to take the perspective or to “put yourself in the shoes of the patient” (Reiss 2010 cited in Ekman and Krasner 2017).

Others support the multidimensional nature of empathy, but indicate slightly different components. Decety and Lamm (2006), for instance, hold that empathy consists of three dimensions: emotion sharing, perspective-taking and emotional regulation. However, this third component differs from the ones stated above, as it involves the capability to both regulate and modulate the empathic response. With regards to this formulation, Shapiro (2011) suggests that we should not ignore and suppress the uprise of emotions, but rather regulate and modulate them.

In line with this reasoning, research in neuroscience has shown that the awareness of a distinction between the experiences of the self and the experiences of others constitutes a crucial aspect of an ideal clinical empathy. Thus, the psychological construct of empathy could be defined as the intersubjective induction process by which emotions are shared *without forgetting what feelings belong to whom* (Decety and Meyer 2008 cited in Ekman and Krasner 2017).

In fact, empathy can only be effective if individuals are able to separate their own feelings from those shared with others, thus having self-awareness and other-awareness (Decety and Lamm 2006). In other words, “to sense the [careseeker] private world ‘as if’ it were your own but without ever losing the ‘as if’ quality—this is empathy and this seems essential to therapy” (Gair 2012, p. 832).

This being said, clinical empathy encloses a detailed experiential and a cognitive understanding of what the patient is feeling. As such, it is neither emotional detachment nor a complete immersion in the other’s experience. Accordingly, and as we will later stress, empathy is “an ongoing double movement of emotional resonance and compassionate curiosity about the meaning of the clinical situation to the patient” (Shapiro 2011, p. 276).

Building upon Geldard and Geldard (2005), we believe that empathy is about “having a togetherness with the [patient]... going on a journey with [patients], listening with sensitivity, matching their every move... walking beside the [patient]” (p. 18). When experienced with self-awareness, clinical empathy shows to be a mutually healing connection with the patient (Kearney et al. 2009 cited in Shapiro 2011); and thus, it is not lived as a detrimental emotional burden.

## Benefits of empathy in patient care

As mentioned, patients have reported the longing for a more human-oriented contact with caregivers. According to research, clinical empathy allows patients to feel respected and validated in their experience of illness (Beckman et al. 1994). Within this framework, there have been attempts to bring the medical approach towards a relationship-centered care model; where the human dimension is central and patients’ experiences are seen as valuable. Here, clinical empathy is considered as a means of helping patients to experience and express emotions (Beach and Inui 2006 cited in Garden 2009).

Research shows that allowing space for a patient’s narrative is neither inefficient nor time-consuming. In fact, giving patients space to express their personal concerns has been shown to take no longer than 90 s and yet to considerably improve patient satisfaction and adherence to treatment (Langewitz et al. 2002 cited in Halpern 2014).

This being said, not only does this type of empathy promote patients’ and physicians’ satisfaction and well-being, but it also plays a role in improving medical outcomes (Bikker et al. 2005; MacPherson et al. 2003; Coulehan et al. 2001; Vermeire et al. 2001; Suchman et al. 1993 cited in Chen et al. 2012). In fact, clinical empathy helps clinicians recognize, understand and accept patients in their suffering. This inclusive understanding is the first step towards alleviating their distress; something that occurs through the process of ‘being moved’ to action (Garden 2009).

### For patients

Through its capacity to cultivate trust, communication, and mutual understanding, clinical empathy has positive impacts on multiple levels. Generally speaking, it has been reported that empathic caregiving improves patient satisfaction as well as clinical outcomes, to name a few (Mercer et al. 2016; Shanafelt et al. 2002; Shapiro et al. 1989 cited in Ekman & Krasner 2017; Derksen et al. 2013; Gleichgerrcht & Decety 2013 cited in Hooker 2015; Hojat et al. 2013; 2011; Rosenthal et al. 2011; Zachariae et al. 2003 cited in Finset 2010; Bertakis et al. 1991; Beckman et al. 1984; Francis et al. 1969 cited in Hojat 2009).

In a study conducted by Zachariae et al. (2003) with oncology patients, high scores of physician’s empathy and consideration resulted in improved patient’s experience of care. Additionally, the scores were associated with increased autonomy, and reduced emotional distress after the *consultatio* (Finset 2010). In another study (Graugaard et al. 2004 cited in Finset 2010), empathy was shown to increase fibromyalgia patients’ satisfaction; in particular among those with difficulties in expressing their own emotions.

Further, it has been reported that one of the biggest limitations of effective medical treatment is patient non-adherence. To this regard, studies have shown that about fifty percent of treatments are not taken as prescribed (Ekman and Krasner 2017); something that can negatively interfere with the patient’s recovery.

According to academic research, empathy may offer a solution to this problem. In fact, empathy in patient care results into positive clinical outcomes, such as patients’ compliance with the medical care plan and engagement in medical recommendations (Ekman and Krasner 2017; Rosenthal et al. 2011; Roter et al. 1998 cited in Halpern 2014; DiMatteo et al. 1986, 1993; Squier 1990; Eisenthal et al. 1979 cited in Hojat 2009).

Additionally, empathy has been shown to facilitate patients’ understanding of treatment options and participation in making decisions concerning therapy. By feeling empowered in the therapeutic process, patients are more inclined to follow their medical treatment plan (Halpern 2014, 2001 cited in Hooker 2015).

Going back to the central role played by trust in the empathic relationship between health practitioner and patient, a meta-analysis of factors that improve adherence to treatment (Roter et al. 1998) illustrates that trust was one of the most important steps towards engagement in medical treatment. In other words, the patient’s feeling of trust was the sense that the physician was genuinely worried about them.

Additionally, other studies (Kim et al. 2004 cited in Halpern 2014) have suggested that patients trust empathic physicians more, and that this increases the effectiveness of medical care. Overall, an empathic relationship leads to better physiological responses and shorter hospitalization (Reiss 2010 cited in Halpern 2011). Such benefits result in lower costs of medical care (Rosenthal et al. 2011; Hojat 2009).

Another factor that leads to reducing costs has been studied by Nightingale et al. (1991). In this study, it was shown that empathic physicians avoided unnecessary costs by ordering fewer laboratory tests and were less inclined to perform invasive and costly interventions, unless strictly necessary.

As mentioned, the current medical culture stigmatizes and does not provide a sufficient framework to prevent medical error. In a study conducted by a group of medical residents training at the Mayo Clinic, error was associated with lower levels of empathy (Ekman and Krasner 2017). Thereby, we may assume that through the use of empathy physicians make fewer errors during the diagnostic procedure. This may occur because empathic physicians are more prone to obtain critical information and deeper insights; something that positively affects the overall quality of care and fosters favorable medical outcomes (Shanafelt et al. 2009 cited in Reiss 2010).

### For caregivers and medical students

Coming to the benefits for physicians and medical students, research has reported a correlation between an empathic approach and health professionals’ personal well-being and job satisfaction (Shanafelt et al. 2012; AAMC 1998 cited in Ekman and Krasner 2017; Larson and Yao 2005 cited in Hooker 2011; Rosenthal et al. 2011; Hojat 2009).

Additionally, empathic communication has been correlated with fewer malpractice claims and fewer patients’ litigations (Rosenthal et al. 2011; Moore et al. 2000; Steward et al. 1999; Levinson et al. 1997; Beckman et al. 1994; Hickson et al. 1992; 1994; Shapiro et al. 1989; Avery 1985 cited in Hojat 2009).

Furthermore, lower empathy may increase the occurrence of medical errors as well as increase the distress experienced by health professionals (Ekman and Krasner 2017). On the same line of reasoning and contrarily to the tendency to regard empathy as unnecessary, when not harmful, higher levels of empathy have been shown to prevent symptoms of burnout (Diorio and Nowaczyk 2019), substance abuse and suicide amongst both physicians and trainees (Shanafelt et al. 2009 cited in Reiss 2010).

## From clinical empathy to empathic concern

We have looked at the benefits that come along with the implementation of what we refer to as clinical empathy. In accordance with academic scholar Halpern (2011) we hold that we should go beyond the mere focus on the debate regarding the different components of empathy and the manner in which they interrelate in empathic interaction.

In this section we attempt to overcome the potential downfalls of the rigid taxonomy surrounding the term ‘empathy’, whereby empathy is dichotomously divided into ‘cognitive’ and ‘emotional’ empathy. This is why we propose the notion of ‘empathic concern’, whereby clinical empathy is understood as the attitude of genuine interest towards the experience of the other, and that results from ‘engaged curiosity’.

In the attempt to address the patient’s need to be valued and understood, Halpern (2011) suggests that clinicians embrace the notion of ‘engaged curiosity’*.* She defines the latter as a “genuine, emotionally engaged interest in learning more about the complexity of the patient’s (and our responsive) emotional points of view” (p. 308); something that we propose a precondition for ‘empathic concern’. Here, ‘concern’ is very different from the said detached concern and translates a mere commitment to heal to a *validation* of the patient’s suffering and needs.

Thereby, empathic concern in the clinical setting derives from a genuine interest in the patient and varies according to each unique experience (Halpern, 2011). In fact, the latter can never be generalized and strictly associated with a specific ‘type’ of empathy. This empathic engagement is reached through *curiosity* about what the patient is specifically concerned about, *non-verbal attunement* and *the effort to imagine* the other’s experience. The health professional is thus led to welcome a deeper and more comprehensive understanding of the patient’s experience.

In fact, there are situations in which it is simply necessary to acknowledge the patient as a human being “with feelings and worth” (Halpern 2011). This means that in many contexts clinicians are not expected to engage in any sort of specific empathy. To this regard, according to Halpern (2011) patients reportedly appreciate their physician’s curiosity even in moments when the caregiver has trouble fully understanding them and what they are going through.

Moreover, clinicians are aware of the fact that they will not completely understand their patients unless they actively try to find out more about the patient’s situated experience; that is, what the patient is needing, seeking or afraid of. This awareness is what allows clinicians to maintain the distinction between the self and the other; something that ultimately avoids unnecessary distress and/or error deriving from over-identification (ibid).

Multiple studies have shown the validity of such an approach. For instance, an observational study conducted by Shanafelt et al. (2005) in 2005 illustrated that when physicians show non-verbal attunement (for example through gestures), patients give fuller stories and are more open to talk about the emotional aspects of their narratives; often critical elements for a correct diagnosis, and thus essential for an effective medical treatment. Similarly, Suchman et al. (1997) reported that, in many cases, patients tell important information to physicians who are not verbally attuned, but who rather show hints of their emotional engagement.

In line with the above-mentioned results, according to Halpern (2014) engaged curiosity can help caregivers avoid clinical mistakes, such as ‘projection’, ‘overidentification’ and ‘naive sympathy’. Projection is the process by which physicians project themselves onto the patients. When this occurs, physicians think that they know what their patient is worried or concerned about; even when, in actuality, this is not the case. It may also happen that clinicians over-empathize with their patient, and thus think that what the latter is going through is similar to what they have experienced.

By doing so, the physician overlooks the fact that individuals may experience the same thing in a different manner, and thereby fall into the trap of overidentification. Both projection and overidentification may give rise to naive sympathy; a superficial emotional engagement that is usually not appreciated by the patient, as it does not confer an adequate importance to the patient’s situation (Halpern 2014).

## Fostering empathic concern in patient care: the role of Narrative Medicine

We have seen how the current medical culture, with its worship of objectivity and emotional detachment, has put aside the importance of acknowledging the human experience of illness. This phenomenon is closely linked to medical training. In fact, the latter has the tendency to neglect the individual of the fundamental aspects of patient care; namely, the human relationship in the context of illness.

In this paper, we have made an attempt to problematize the rigid taxonomy and dissection of the notion of empathy that is still prominent in existing scholarship. By questioning the widespread dichotomous distinction between cognitive and affective empathy, we highlighted the potential of what is referred to as clinical empathy as a more *holistic* human capacity.

According to Halpern (2014), one of the basic elements of patient care is the caregiver’s ability to make an effort to listen to the patient’s unique experience with illness and sensitively communicate this understanding to them.

Following this line of reasoning, we illustrated Halpern’s notion of engaged curiosity as a precondition for what we propose as a new understanding of clinician empathy; namely, empathic concern. The latter being an approach that gives value to a genuine interest in welcoming, in acknowledging and in responding to the emotions of those suffering. In this sense, empathic concern calls for an interest on behalf of the clinician that allows patients to feel recognized, understood and accepted in their hardship.

This approach presents many analogies with ‘Narrative Medicine’, a medical approach that “fortifies the clinical practice with the narrative competence to recognize, absorb, interpret and to be moved by the stories of illness” (Charon 2007, p. 1265). In this sense, Narrative Medicine represents a form of medical practice that brings to light and validates the stories of the ill. Illness unfolds in stories and it should be the medical practitioner’s concern to listen and to honor those narratives, so as to recognize and legitimize the basic dignity of the patient.

Narrative Medicine is a person-centered approach, as it focuses on active listening and engaged communication, which elicit the patient’s perspective and experience (Finset 2010; Charon 2004). In this sense, Narrative Medicine represents one of the attempts to rehumanize medicine and to address the issues that can be found in Flexner’s model, as discussed in the section concerning the current western medical culture.

As a more holistic and comprehensive medical-care model, Narrative Medicine promotes self-awareness and other-awareness. In this sense, it constitutes a ‘middle ground’ between medical practitioners and patients, as it allows the former to take the other’s perspective and explore a subjective experience of illness through the facilitation of the patient’s expression (Johna and Rahman 2011). This occurs thanks to the recognition of empathic opportunities; in other words, hints and cues that can elicit empathic concern towards the patient (Eide et al. 2004 cited in Finset 2010).

As mentioned, the core values of Narrative Medicine can also be reached by fortifying the current medical training with narrative practices. In fact, something that is central to narrative competence is the capacity for self-reflection, awareness and interpretation of emotional responses of the other, and the development of insight and sensitivity to be moved and act accordingly to the patient’s needs, being these emotional or physiological.

To this regard, in a 2001 study Charon (2001) has shown that clinicians who learn to write their patient’s narratives display more empathic concern for their patients in subsequent clinical work. Following this line of reasoning, it has been shown that writing exercises and the study of literary texts can help caregivers to keep their focus on, as well as to value their patient’s unique experience of illness (Garden 2009).

Additionally, Bonvicini et al. (2009) have reported the results of a randomized controlled study, according to which by taking part in a communication training practice, physicians significantly improved their empathic expression when interacting with a patient. By welcoming and acknowledging their patient’s suffering, health professionals carry out one of the basic tasks, that is welcoming the human experience of suffering and respecting the individual in their entirety.

## Conclusion

As we have seen, the definition of empathy and its application in the medical field are widely debated among scholars. In this critical review, we attempted to present some of the different views on the topic of debate. We then shed light on the dehumanization of the medical system (Haque and Waytz 2012), and its negative impacts on the quality of healthcare and on the wellbeing of both patients and health practitioners. Further, we illustrated how such a phenomenon is reinforced in and by medical education and training.

After analyzing the discussion on the very essence of empathy and then, on its application within the clinical setting, we have come to the conclusion that such debate leads to an excessively rigid taxonomy, as it divides the cognitive and the emotional dimensions of empathy.

Following this, we attempted to overcome potential downfalls of the rigid taxonomy surrounding the term ‘empathy’, whereby empathy is dichotomously divided into ‘cognitive’ and ‘emotional’ empathy. In this sense, we believe that clinical empathy should be conceived of as ‘empathic concern’; an attitude that configures itself as a genuine interest towards the other, and that results from Halpern’s notion of ‘engaged curiosity’. The latter being understood as a posture that gives value to a genuine interest in welcoming and acknowledging the complexity in the experience of those suffering (Halpern 2011).

In our view, empathic concern would avoid some of the misleading discussions that revolve around the very definition of clinical empathy. Further, it would redirect the focus of research towards a more multidimensional conception of empathy in patient care and beyond the taxonomy of empathy. In fact, empathic concern bears both the more cognitive perspective and the emotional engagement and responsiveness dimensions of empathy. This is how a mere commitment to heal can translate into a *validation* of the patient’s suffering and needs.

Finally, we stressed the potential link between Halpern’s engaged curiosity and the practice of Narrative Medicine, as defined by Rita Charon in *Narrative Medicine: Honoring the Stories of Illness* (2006). We see the latter as a practical solution to address the lack of empathic concern in the caregiver-patient interaction. To this regard, we call for further research in the field of Narrative Medicine and, particularly, in its role in teaching and promoting engaged curiosity, which we identified as a precondition for empathic concern.
